# Population and Energy Transfer Dynamics in an Open Excitonic Quantum Battery

**DOI:** 10.3390/molecules29040889

**Published:** 2024-02-17

**Authors:** Zhe Liu, Gabriel Hanna

**Affiliations:** Department of Chemistry, University of Alberta, Edmonton, AB T6G 2G2, Canada; zhe17@ualberta.ca

**Keywords:** quantum battery, exciton dynamics, quantum energy storage, dark state

## Abstract

In a previous study, we proposed an open quantum network model of a quantum battery (QB) that possesses dark states owing to its structural exchange symmetries. While in a dark state, the QB is capable of storing an exciton without any environment-induced population losses. However, when the structural exchange symmetry is broken, the QB begins to discharge the exciton towards its exit site. In this article, we start by demonstrating that this QB is not only loss-free with respect to exciton population during the storage phase, but also with respect to the QB energy. We then explore the exciton population and energy transfer dynamics of the QB during the discharge phase over a wide range of site energies, bath temperatures, and bath reorganization energies. Our results shed light on how to optimize the QB’s population and energy transfer dynamics for different purposes.

## 1. Introduction

A quantum battery (QB) is a quantum system that can store and release energy as needed. Over the past decade, several types of QBs have been proposed, demonstrating advantages with respect to charging speed [1,2,3,4,5,6,7,8,9] and work extraction [5,7,10,11,12,13,14] over the classical analogues. In practice, a QB may interact with an environment, which could lead to losses that negatively impact its performance. Thus, any theoretical study should treat the QB as an open quantum system [15,16,17]. Previously, open quantum network (OQN) models, in which the network sites (representing quantum systems) may be coupled to each other and to dissipative/decohering environments, have been used to study the dynamics of open QBs [5,6,8,18].

Over the years, a number of ways of protecting a quantum system from its environment have been proposed. One well-known approach is based on the use of a decoherence-free subspace (DFS) [19,20]—a subspace of the Hilbert space in which the dynamics is purely unitary. While in a DFS, the system’s dynamics is dissipationless and decoherence-free despite the coupling to an environment. As has been shown in Refs. [21,22,23], if there exists a unitary operator that commutes with all elements in the system’s master equation exists, then the system will possess invariant subspaces. Among such subspaces, the one-dimensional subspaces are DFSs because the dynamics maps them onto themselves. Owing to this property, DFSs have many potential applications in quantum information and quantum computing [24,25,26,27,28,29].

Recently, researchers have used dark states living in DFSs for stabilizing and enhancing the performance of QBs and other OQNs [18,30,31,32]. In particular, we proposed an OQN model of a QB with site exchange symmetries that support the existence of dark states localized on the bulk sites of the network [18]. We showed that it is possible to store an exciton in one of these dark states, without any exciton population transfer to the two surface sites which are connected to thermal baths at equal temperatures. In this way, it was possible to protect the QB from environment-induced excitation energy losses. Moreover, we showed that by attaching an additional bath to break the exchange symmetry of the QB, it is possible to discharge the exciton towards the designated exit site to be ultimately harnessed by a sink.

Although it was shown that our QB model is capable of operating as an *excitonic* QB (i.e., a QB that stores and discharges excitons), the question of whether or not the model is capable of operating as an *energy* battery (i.e., a QB whose energy does not decrease significantly during the storage phase and discharges energy to a sink with minimal loss to the baths) remained to be explored. Therefore, in this study, in addition to monitoring the exciton population dynamics, we calculate the various contributions to the total energy of the system during both the storage and discharge phases for a wide range of bath temperature gaps, bath reorganization energies, and site energies. The aim of the study is to identify the conditions which optimize the performance of the QB model for its use as an excitonic QB, energy QB, or both.

The paper is organized as follows. First, in Section 2, we introduce the open QB model. In Section 3, we explain how the dynamics of the model are simulated and how the populations/energies are calculated. In Section 4, we present the time-dependent populations/energies of the model for different bath temperatures, bath reorganization energies, and site energies. The performance of the QB under the various conditions is also discussed. In Section 5, we summarize our findings.

## 2. Open QB Model

**Figure 1:**
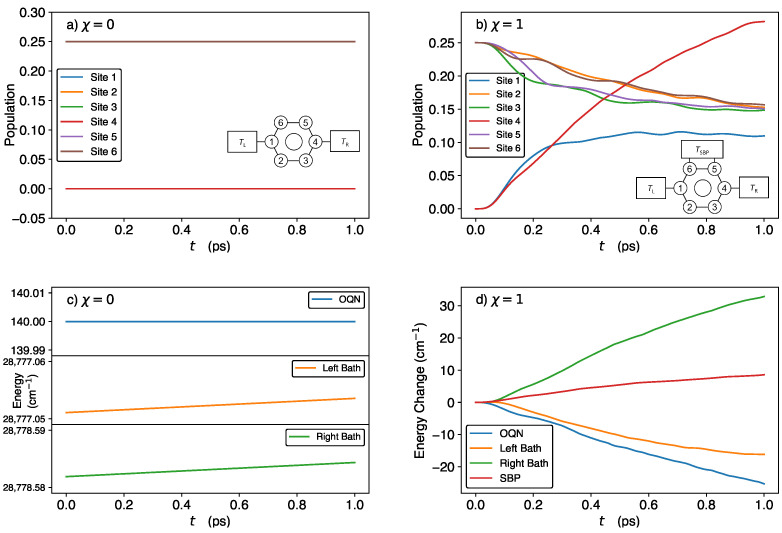
Time-dependent site populations (**upper panels**) and energies (**lower panels**) of the OQN, SBP, and baths in the storage (**left panels**) and discharge (**right panels**) phases. In panel (**d**), energy changes are calculated by subtracting the initial value of the energy from the value at each time. The results were generated using the following parameter set: $E_{1}=250\;{\mathrm{cm}}^{-1},E_{i\in \{2,3,5,6\}}=200\;{\mathrm{cm}}^{-1},E_{4}=0\;{\mathrm{cm}}^{-1}$, $h=-60\;{\mathrm{cm}}^{-1}$, $T_{L}=T_{R}=300$ K, $T_{p}=300$ K, $\lambda_{b}=35\;{\mathrm{cm}}^{-1}$, $\lambda_{p}=10\;{\mathrm{cm}}^{-1}$, $\omega_{c}=\omega_{p}=106\;{\mathrm{cm}}^{-1}$, $\omega_{max}=50\omega_{c}$, and M=100.

Following Ref. [18], we consider the same six-site para-benzene-shaped OQN (the numbering of the sites is depicted in Figure 1b). Setting ℏ=1, the Hamiltonian of the closed network is given by

(1) $${\hat{H}}_{N}=\sum_{n=1}^{6}E_{n}\left|n\rangle \langle n\right|+h\sum_{\langle n,m\rangle }|n\rangle \langle m|,$$

where |n〉 corresponds to a singly excited state localized on site *n*, $E_{n}$ is the energy of site *n*, *h* is the electronic coupling strength between sites *n* and *m*, and 〈n,m〉 denotes that a cyclic summation over nearest-neighbour sites is performed. To construct the OQN, the two para-sites of the network are coupled to thermal baths, each composed of a set of independent harmonic oscillators. The sum of the bath and network–bath coupling Hamiltonians is given by

(2) $${\hat{H}}_{B}+{\hat{H}}_{NB}\;=\;\frac{1}{2}\sum_{n\in SSs}\sum_{j}^{M}\left[{\hat{P}}_{n,j}^{2}+\omega_{n,j}^{2}{\left({\hat{R}}_{n,j}-\frac{C_{n,j}}{\omega_{n,j}^{2}}\left|n\rangle \langle n\right|\right)}^{2}\right],$$

where *M* is the number of harmonic oscillators in each bath, ${\hat{P}}_{n,j}$ and ${\hat{R}}_{n,j}$ are the mass-weighted momentum and position operators, respectively, of the *j*th oscillator with frequency $\omega_{n,j}$, and $C_{n,j}$ is the network–bath coupling strength between the *n*th site and the *j*th oscillator. The sites coupled to the baths are referred to as surface sites (SSs) while the remaining sites are referred to as bulk sites (BSs). In this study, we take the site energies of all BS to be equal to a value $E_{BS}$, while considering different values of site energies of the SS.

The network defined by ${\hat{H}}_{N}$ and the aforementioned site energies possesses the following unitary symmetry operator [33]

(3) $$\hat{\Pi }\;=\;|1\rangle \langle 1|+\left|4\rangle \langle 4\right|+\left|2\rangle \langle 6\right|+\left|3\rangle \langle 5\right|+h.c.,$$

which satisfies

(4) $$\left[\hat{\Pi },{\hat{H}}_{N}\right]=0,\;\;\left[\hat{\Pi },{\hat{H}}_{NB}\right]=0\;\;\;\;\forall n\in \mathrm{SSs}.$$

As a result, $\hat{\Pi }$ shares the same eigenstates as ${\hat{H}}_{N}$. Due to the existence of this symmetry operator, the system possesses two DFSs with the following DSs ($|\psi_{\alpha }\rangle$) and eigenvalues ($u_{\alpha }$) [22,34]

(5) $$\begin{array}{ccc} |\psi_{1}\rangle & = & \frac{1}{2}\left(|5\rangle +|6\rangle -|2\rangle -|3\rangle \right),\;\;\;\;u_{1}=E_{BS}+h\\ |\psi_{2}\rangle & = & \frac{1}{2}\left(|3\rangle +|6\rangle -|2\rangle -|5\rangle \right),\;\;\;\;u_{2}=E_{BS}-h \end{array}$$

If the system is initialized in the dark state $|\psi_{\alpha }\rangle$, it will undergo dissipationless dynamics, i.e., the dark state will be invariant under the effect of the evolution operator of the composite system and the site populations will be

(6) $$\langle {\hat{P}}_{nn}\left(t\right)\rangle =\langle {\hat{P}}_{nn}\left(0\right)\rangle =\left\{\begin{array}{c} 0,\;\;\;\forall n\in \mathrm{SSs},\\ \frac{1}{4},\;\;\;\forall n\in \mathrm{BSs}, \end{array}\right.$$

where ${\hat{P}}_{nn}=\left|n\rangle \langle n\right|$ is the projection operator corresponding to site *n* and 〈·〉 denotes an ensemble average. Because an exciton can be stored indefinitely in this state, this phase is termed the *storage phase*.

When a symmetry-breaking perturbation (SBP) is connected to the OQN, the symmetry operator and DSs no longer persist and the QB may begin to discharge a stored exciton. In this study, this is achieved by attaching a bath of *M* harmonic oscillators to sites 2 and 3 simultaneously. The sum of the SBP and network-SBP coupling Hamiltonians is

(7) $${\hat{H}}_{P}+{\hat{H}}_{NP}\;=\;\frac{1}{2}\sum_{k}^{M}\left[{\hat{p}}_{k}^{2}+\Omega_{k}^{2}{\left({\hat{r}}_{k}-\frac{\gamma_{k}}{\Omega_{k}^{2}}\hat{S}\right)}^{2}\right],$$

where ${\hat{p}}_{k}$, ${\hat{r}}_{k}$, $\Omega_{k}$, and $\gamma_{k}$ are the momentum operator, position operator, frequency, and network–SBP coupling strength of the *k*th oscillator, respectively, and $\hat{S}=\left|2\rangle \langle 2\right|+\left|3\rangle \langle 3\right|$. This phase is termed the *discharge phase*.

## 3. Simulation Details

Due to the large number of degrees of freedom in the composite system (i.e., QB, thermal baths, and SBP), a fully quantum dynamical simulation of the composite system would be computationally expensive. Thus, following Ref. [18], we use a mixed quantum–classical dynamics method known as “Deterministic evolution of coordinates with initial decoupled equations” (DECIDE) [35,36], which treats the OQN quantum mechanically and the baths and SBP in a classical-like way. Previously, the DECIDE method has been successfully applied to a host of model systems over a large range of parameter regimes [35,37,38,39,40], and is, therefore, expected to produce reliable results in this study. That being said, DECIDE may yield inaccurate results for systems with very slow thermal baths (i.e., when the bath cut-off frequency is much smaller than the subsystem energy gaps) or with very low bath temperatures, neither of which is the case in the present study.

To apply the DECIDE method, we must first apply the Wigner transform [41] to the bath and SBP degrees of freedom. The resulting partially Wigner-transformed Hamiltonian of the composite system is

(8) $${\hat{H}}_{W}\;=\;{\hat{H}}_{N}+{\hat{H}}_{NB}\left(\left\{R_{n,j}\right\}\right)+H_{B}\left(\left\{P_{n,j}\right\},\left\{R_{n,j}\right\}\right)+\chi \left[H_{P}\left(\left\{p_{k}\right\},\left\{r_{k}\right\}\right)+{\hat{H}}_{NP}\left(\left\{r_{k}\right\}\right)\right],$$

where $\left\{R_{n,j},P_{n,j}\right\}$ and $\left\{r_{k},p_{k}\right\}$ are the position and momentum variables of the baths and SBP, respectively. The parameter χ is equal to 1 when the SBP is attached to the OQN and 0 otherwise. The coordinates of the OQN are taken to be ${\hat{P}}_{nm}=\left|n\rangle \langle m\right|$, while the coordinates of the baths and SBP are their positions and momenta. According to the DECIDE method, the coupled equations of motion for all of the coordinates are given by [18]

(9) $$\begin{array}{ccc} \frac{d}{dt}P_{nm}^{\beta \beta^{\prime }}\left(t\right) & = & i{\left[\sum_{l=1}^{6}V_{ln}{\hat{P}}_{lm}\left(t\right)-\sum_{v=1}^{6}V_{mv}{\hat{P}}_{nv}\left(t\right)\right]}^{\beta \beta^{\prime }}\\ & & -\frac{i}{2}\sum_{j}C_{n,j}{\left(R_{n,j}\left(t\right){\hat{P}}_{nm}\left(t\right)+{\hat{P}}_{nm}\left(t\right)R_{n,j}\left(t\right)\right)}^{\beta \beta^{\prime }}\left(\delta_{n,1}+\delta_{n,4}\right)\\ & & +\frac{i}{2}\sum_{j}C_{m,j}{\left(R_{m,j}\left(t\right){\hat{P}}_{nm}\left(t\right)+{\hat{P}}_{nm}\left(t\right)R_{m,j}\left(t\right)\right)}^{\beta \beta^{\prime }}\left(\delta_{m,1}+\delta_{m,4}\right)\\ & & -\chi \frac{i}{2}\sum_{k}C_{k}{\left(R_{k}\left(t\right){\hat{P}}_{nm}\left(t\right)+{\hat{P}}_{nm}\left(t\right)R_{k}\left(t\right)\right)}^{\beta \beta^{\prime }}\left(\delta_{n,2}+\delta_{n,3}\right)\\ & & +\chi \frac{i}{2}\sum_{k}C_{k}{\left(R_{k}\left(t\right){\hat{P}}_{nm}\left(t\right)+{\hat{P}}_{nm}\left(t\right)R_{k}\left(t\right)\right)}^{\beta \beta^{\prime }}\left(\delta_{m,2}+\delta_{m,3}\right),\\ \frac{d}{dt}R_{n,j}^{\beta \beta^{\prime }}\left(t\right) & = & P_{n,j}^{\beta \beta^{\prime }}\left(t\right),\\ \frac{d}{dt}P_{n,j}^{\beta \beta^{\prime }}\left(t\right) & = & -\omega_{n,j}^{2}R_{n,j}^{\beta \beta^{\prime }}\left(t\right)+C_{n,j}P_{nn}^{\beta \beta^{\prime }}\left(t\right)\left(\delta_{n,1}+\delta_{n,4}\right),\\ \frac{d}{dt}r_{k}^{\beta \beta^{\prime }}\left(t\right) & = & p_{k}^{\beta \beta^{\prime }}\left(t\right),\\ \frac{d}{dt}p_{k}^{\beta \beta^{\prime }}\left(t\right) & = & -\Omega_{k}^{2}r_{k}^{\beta \beta^{\prime }}\left(t\right)+\chi \gamma_{k}{\left({\hat{P}}_{22}+{\hat{P}}_{33}\right)}^{\beta \beta^{\prime }}\left(t\right), \end{array}$$

where $V_{nn}=E_{n}+\sum_{j=1}^{M}C_{n,j}^{2}/\left(2\omega_{n,j}^{2}\right)\left(\delta_{n,1}+\delta_{n,4}\right)+\chi \sum_{k=1}^{M}\gamma_{k}^{2}/\left(2\Omega_{k}^{2}\right)\left(\delta_{n,2}+\delta_{n,3}\right)$, and when n≠m, $V_{nm}=h$ for |n−m|=1 and 0 otherwise. Here, β labels an arbitrary basis state, i.e., the matrix element of ${\hat{P}}_{nm}$ is given by $P_{nm}^{\beta \beta^{\prime }}=\langle \beta |{\hat{P}}_{nm}|\beta^{\prime }\rangle$. In this work, {|β〉}={|1〉,|2〉,⋯,|6〉}.

We assume the initial state of the composite system to be factorized, i.e., ${\hat{\rho }}_{tot}\left(0\right)={\hat{\rho }}_{N}\left(0\right)\rho_{B,W}\left(0\right)\rho_{P,W}\left(0\right)$, where ${\hat{\rho }}_{N}\left(0\right)$ is the initial density operator of the network, and $\rho_{B,W}\left(0\right)$ and $\rho_{P,W}\left(0\right)$ are the initial Wigner-transformed densities of the heat baths and SBP, respectively (N.B.: $\rho_{P,W}\left(0\right)$ is omitted when the OQN is not attached to the SBP). The initial state of the network is taken to be the dark state ${\hat{\rho }}_{N}\left(0\right)=|\psi_{1}\rangle \langle \psi_{1}|$, where $|\psi_{1}\rangle$ is defined in Equation (5). The initial values of the OQN coordinates are always taken to be $P_{nm}^{\beta \beta^{\prime }}=\delta_{\beta ,n}\delta_{m,\beta^{\prime }}$. The bath oscillators are initialized in the thermal equilibrium state given by (setting $k_{B}=1$) [42]

(10) $$\rho_{B,W}\left(0\right)=\prod_{n=1,4}\prod_{j=1}^{M}\frac{\tanh(\beta \omega_{n,j}/2)}{\pi }\exp\left[-\frac{2\tanh(\beta \omega_{n,j}/2)}{\omega_{n,j}}\left(\frac{P_{n,j}^{2}}{2}+\frac{\omega_{n,j}^{2}R_{n,j}^{2}}{2}\right)\right],$$

where $\beta =1/k_{B}T$ is the inverse temperature. The oscillators of the SBP are also initialized in a thermal equilibrium state with an analogous form. The initial positions and momenta of the bath and SBP oscillators are sampled from Equation (10) and its analog for the SBP, respectively. The system–bath and system–SBP couplings are characterized by a Debye–Drude spectral density, i.e., $J\left(\omega_{n,j}\right)=2\lambda_{b}\frac{\omega_{n,j}}{\omega_{n,j}^{2}+1}$. In this work, the spectral density is discretized to yield the following expressions for the coupling strengths $C_{n,j}$ and frequencies $\omega_{n,j}$ [43,44]:

(11) $$\omega_{n,j}=\tan\left(j\arctan\left(\omega_{max}/\omega_{c}\right)/M\right)\omega_{c},$$

(12) $$C_{n,j}\;=\;2\sqrt{\lambda_{b}\arctan\left(\omega_{max}/\omega_{c}\right)/\left(\pi M\right)}\omega_{n,j},$$

where $\lambda_{b}$ is the bath reorganization energy and $\omega_{c}$ is the bath cut-off frequency [45].

Previously, the fourth-order Runge–Kutta method was used to integrate the DECIDE equations of motion in Equation (9), yielding conserved total populations for the system under study [18,35]. However, in this work, we found that a combination of a smaller time step and a higher order integrator is needed for good energy conservation and more accurate calculations of the various contributions to the total energy. High-order methods such as the eighth-order Runge–Kutta method [46] can yield accurate results with a relatively large time step, but it contains many integration stages which increase the simulation time drastically. Considering the trade-off between time step and number of integration steps, we employed the sixth-order Runge–Kutta method in this work. Using this integrator with a time step of 0.16 fs, the total energy drift is less than $10^{-2}\;{\mathrm{cm}}^{-1}$ over a 1 ps trajectory [see Section S1 of the Supporting Information (SI)].

The time-dependent population of site *n* is calculated via an ensemble average of the projection operator ${\hat{P}}_{nn}$, viz.,

(13) $$\langle {\hat{P}}_{nn}\left(t\right)\rangle =\sum_{\beta \beta^{\prime }}\int \;dX\left(0\right)P_{nn}^{\beta \beta^{\prime }}\left(t\right)\rho_{N}^{\beta^{\prime }\beta }\left(0\right)\rho_{E,W}\left(0\right),$$

where $X\left(0\right)=(\left\{R_{n,j}\right\},\left\{P_{n,j}\right\},\left\{r_{k}\right\},\left\{p_{k}\right\})$ are the initial coordinates of the baths and SBP. Similarly, the average total energy of the composite system is

(14) $$\langle E_{tot}\left(t\right)\rangle =\sum_{\beta \beta^{\prime }}\int \;dX\left(0\right){\hat{H}}_{W}^{\beta \beta^{\prime }}\left(t\right)\rho_{N}^{\beta^{\prime }\beta }\left(0\right)\rho_{E,W}\left(0\right).$$

In the above equations, $\rho_{E,W}=\rho_{B,W}$ in the absence of the SBP and $\rho_{E,W}=\rho_{B,W}\rho_{P,W}$ when the SBP is attached to the QB. Using our numerical results, we have verified that $\sum_{n=1}^{6}\langle {\hat{P}}_{nn}\left(t\right)\rangle \approx 1$ (i.e., population conservation) and $\frac{d}{dt}\langle E_{tot}\left(t\right)\rangle \approx 0$ (i.e., energy conservation). For the purposes of our analysis, the total energy of the composite system may be decomposed into the following contributions: OQN energy ($E_{N}$), bath energy ($E_{NB}+E_{B}$), and SBP energy ($E_{NP}+E_{P}$). All simulation results are averaged over 10,000 trajectories, which ensures that the error bars are much smaller than the symbols in the figures.

## 4. Results and Discussion

### 4.1. Exciton Storage and Discharge

We start by considering the time-dependent site populations and energies of the OQN, baths, and SBP in the storage (χ=0) and discharge (χ=1) phases, using the parameter values from Ref. [18], viz., $E_{1}=250\;{\mathrm{cm}}^{-1},E_{i\in \{2,3,5,6\}}=200\;{\mathrm{cm}}^{-1},E_{4}=0\;{\mathrm{cm}}^{-1}$ (N.B.: $E_{4}$ is smaller than the energies of the remaining sites because site 4 is designated as the exit site, which in practice would be attached to a sink that captures the exciton/energy), $h=-60\;{\mathrm{cm}}^{-1}$, $T_{L}=T_{R}=300$ K, $T_{p}=300$ K, $\lambda_{b}=35\;{\mathrm{cm}}^{-1}$, $\lambda_{p}=10\;{\mathrm{cm}}^{-1}$, $\omega_{c}=\omega_{p}=106\;{\mathrm{cm}}^{-1}$, $\omega_{max}=50\omega_{c}$, and M=100.

In Figure 1a, we see that the BS and SS populations remain constant at 0.25 and 0, respectively, during the storage phase. Simultaneously, as seen in Figure 1c, the OQN, left-bath, and right-bath energies remain constant (the minor deviations in the bath energies are attributed to numerical errors). Thus, our QB model is capable of perfectly storing both population and energy during the storage phase, as predicted by the theory. In addition, as shown in Section S3 of the SI, the coherences remain constant during the storage phase.

After attaching the SBP, the populations of sites 1 and 4 increase from 0 while the remaining site populations decrease from 0.25 over the 1 ps time period, as seen in Figure 1b. At t=1 ps, the population of site 4 (i.e., the exit site) is greater than that of site 1 and the BS. (The corresponding time-dependent coherences are shown in Figure S2 of the SI). In Figure 1d, we plot the changes in the OQN, left-bath, right-bath, and SBP energies with respect to their initial values. As can be seen, the OQN and left-bath energies decrease while the SBP and right-bath energies increase. Thus, during the discharge phase, energy flows from the OQN and left bath into the right bath and SBP, with considerably more energy flowing to the right bath than to the SBP.

To further analyze the OQN energy ($E_{N}$) change in the discharge phase, we decompose it into the on-site energy and exchange energy, corresponding to the terms $\sum_{n=1}^{6}E_{n}\left|n\rangle \langle n\right|$ and $h\sum_{\langle n,m\rangle }\left|n\rangle \langle m\right|$, respectively, in Equation (1). The change in the exchange energy is therefore given by

(15) $$\Delta E_{\mathrm{exch}}\left(t\right)=h\sum_{\langle n,m\rangle }\left[\langle {\hat{P}}_{nm}\left(t\right)\rangle -\langle {\hat{P}}_{nm}\left(0\right)\rangle \right].$$

At t=0, only $\langle {\hat{P}}_{23}\rangle$, $\langle {\hat{P}}_{32}\rangle$, $\langle {\hat{P}}_{56}\rangle$, and $\langle {\hat{P}}_{65}\rangle$ are non-zero (since the OQN is initialized in the dark state $|\psi_{1}\rangle$). Their values decrease from 0.25 to ≈0.07 over the course of 1 ps (Figure S4), which causes $\Delta E_{\mathrm{exch}}$ to increase. For the dynamics displayed in Figure 1b,d, $\Delta E_{\mathrm{exch}}$ increases by $25.59\;{\mathrm{cm}}^{-1}$ over the course of 1 ps (as calculated by Equation (15)). In fact, $\Delta E_{\mathrm{exch}}$ increases for all of the parameter regimes studied herein. Considering the values of $\langle {\hat{P}}_{nn}\left(0\right)\rangle$ and the fact that $\sum_{n=1}^{6}{\langle \hat{P}}_{nn}\left(t\right)\rangle =1$, the on-site energy change is (setting all BS energies to be equal, i.e., $E_{i\in \{2,3,5,6\}}=E_{BS}$)

(16) $$\begin{array}{ccc} \Delta E_{\mathrm{on}\text{-}\mathrm{site}}\left(t\right) & = & \sum_{n=1}^{6}E_{n}\langle {\hat{P}}_{nn}\left(t\right)\rangle -\sum_{n=1}^{6}E_{n}\langle {\hat{P}}_{nn}\left(0\right)\rangle \\ & = & \left(E_{1}-E_{BS}\right)\langle {\hat{P}}_{11}\left(t\right)\rangle +\left(E_{4}-E_{BS}\right)\langle {\hat{P}}_{44}\left(t\right)\rangle . \end{array}$$

From this expression, we see that the on-site energy change depends only on the time-dependent populations of the SS and the energy differences $E_{1}-E_{BS}$ and $E_{4}-E_{BS}$ (rather than the absolute values of $E_{1}$ and $E_{4}$). [Indeed, shifting all of the site energies by the same constant results in the same dynamics during the discharge phase]. When site 4 is chosen to be the exit site (i.e., $E_{4}<E_{BS}<E_{1}$), a higher site 4 population will lead to a more negative on-site energy change. In fact, for the dynamics displayed in Figure 1b,d, $\Delta E_{\mathrm{on}\text{-}\mathrm{site}}$ decreases by $50.88\;{\mathrm{cm}}^{-1}$ over the course of 1 ps (as calculated by Equation (16)), which is greater than the increase in $\Delta E_{\mathrm{exch}}$. This explains the decrease in the OQN energy observed in Figure 1d.

### 4.2. Effect of Bath Temperature

We next investigate the effect of varying the bath temperature gap on the site populations and energy flow in the QB, in an effort to find parameter sets that maximize the population of the exit site and minimize the energy loss of the OQN. To simplify our exploration of the parameter space, we vary one parameter at a time while keeping the remaining parameters fixed. First, we fix the temperature of the left bath at 300 K and vary the temperature of the right bath, while keeping the OQN, SBP, and remaining bath parameters unchanged.

**Figure 2:**
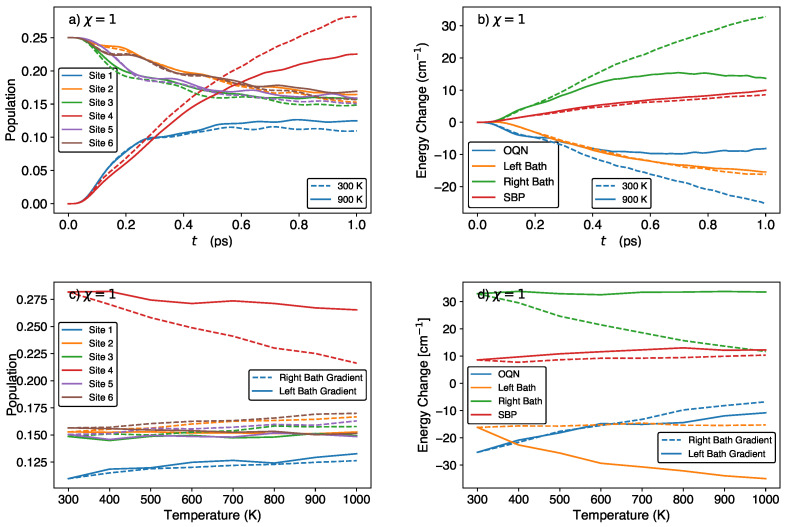
Site populations (**left panels**) and energy changes (**right panels**) in the discharge (χ=1) phase for different bath temperature gradients. (**a**,**b**) Time-dependent site populations and energy changes for right bath temperatures of 300 K (solid lines) and 900 K (dashed lines), with $T_{L}=300$ K. (**c**,**d**) Site populations and energy changes after 1 ps for different right bath temperatures and a left bath temperature of 300 K (denoted by right bath gradient), and different left-bath temperatures and a right-bath temperature of 300 K (denoted by left bath gradient).

In Figure 2a,c, we see that increasing the right bath temperature leads to a decrease in the population of site 4 and relatively small increases in the populations of the remaining sites. As for the energy changes (see Figure 2b,d), the OQN and left bath lose energy, but the energy loss becomes smaller and remains relatively constant, respectively, with increasing right bath temperature. On the other hand, the SBP and right bath gain energy, but the energy gain remains relatively constant and becomes smaller, respectively, with increasing right-bath temperature. When we fix the temperature of the right bath at 300 K and increase the temperature of the left bath, the behaviours of the site populations are similar to those observed in the case when the temperature of the right bath is increased, except for that of site 4 which now exhibits a substantially smaller decrease (see Figure 2c). As for the energy changes (see Figure 2d), the behaviours of the SBP and OQN energies are similar to those observed in the case when the temperature of the right bath is varied. However, the bath energies are significantly different, with the energy changes in the left and right baths becoming more negative and remaining relatively constant, respectively, with increasing left-bath temperature.

Increasing the bath temperature (or decreasing β) will increase the width of the initial Wigner distribution in Equation (10). This increase is particularly significant for oscillators with low frequencies. It can be verified both analytically and numerically that the ensemble averages of the initial bath kinetic energy, $\sum_{j}^{M}P_{n,j}^{2}$, and bath potential energy, $\sum_{j}^{M}\omega_{n,j}^{2}R_{n,j}^{2}$, grow linearly with increasing temperature. For example, increasing the temperature of a bath from 300 K to 600 K to 900 K will increase its kinetic energy from ≈1.43 × $10^{4}$ eV to ≈2.38 × $10^{4}$ eV to ≈3.37 × $10^{4}$ eV.

**Figure 3:**
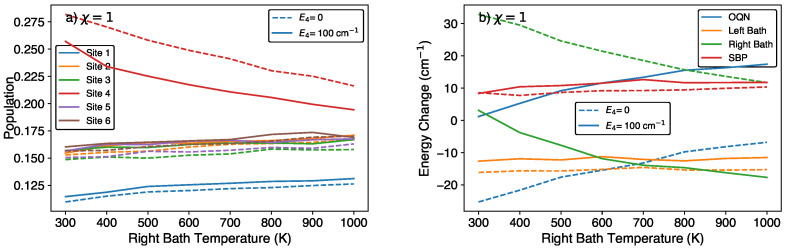
(**a**) Site populations and (**b**) energy changes after 1 ps in the discharge phase for different right-bath temperatures and site 4 energies, with $E_{1}=250\;{\mathrm{cm}}^{-1},E_{i\in \{2,3,5,6\}}=200\;{\mathrm{cm}}^{-1}$, and $T_{L}=300$ K.

As seen in Figure 2d, increasing the left bath or right bath temperature will lead to similar amounts of additional energy transfer out of the bath. This temperature-driven increase in energy transfer to the OQN is relatively independent of the other parameters. As seen in Figure 3, for different site 4 energies, increasing the right bath temperature by a given amount leads to roughly the same increase in energy being transferred from the bath to the OQN. In this way, one can decrease the OQN energy loss by increasing the temperature of any bath. Finally, varying the temperature of a given bath has a larger effect on the site connected to it. For example, if we increase the left bath temperature while keeping the right bath temperature constant, there will be a larger change in the site 1 population than the site 4 population (see Figure 2). These results suggest that one could design a QB that minimizes the OQN energy loss while maintaining a relatively large site 4 population.

### 4.3. Effect of Bath Reorganization Energy

**Figure 4:**
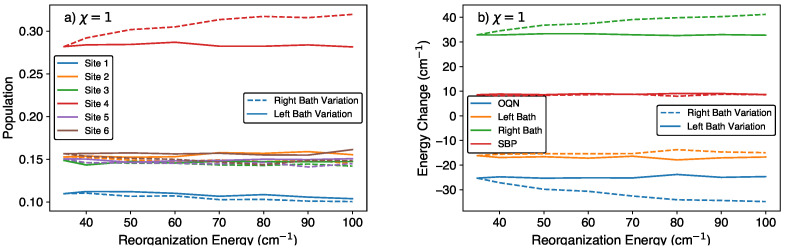
(**a**) Site populations and (**b**) energy changes in the discharge phase after 1 ps for different right-bath reorganization energies and a left-bath reorganization energy of $35\;{\mathrm{cm}}^{-1}$ (denoted by right-bath variation), and different left-bath reorganization energies and a right-bath reorganization energy of $35\;{\mathrm{cm}}^{-1}$ (denoted by left-bath variation).

Next, we vary the bath reorganization energy $\lambda_{b}$. From Figure 4, we see that increasing the right-bath reorganization energy causes the site 1/4 population to decrease/increase, the right-bath energy to increase, and the system to lose more energy; the populations of the remaining sites, left-bath energy, and SBP energy remain relatively constant. On the other hand, we see that increasing the left-bath reorganization energy does not have a significant impact on the site population and energy changes.

As seen in Equation (12), increasing the bath reorganization energy $\lambda_{b}$ will increase the coupling strength $C_{n,j}$. For example, if we increase $\lambda_{b}$ from $35\;{\mathrm{cm}}^{-1}$ to $70\;{\mathrm{cm}}^{-1}$, the coupling strength for each oscillator in the bath will increase by a factor of $\sqrt{2}$. Increasing the coupling strengths increases the magnitudes of the coupling terms in the equations of motion, which translates into faster energy transfer between the system and bath. As seen in Figure 4b, increasing the energy transfer rate of the right bath results in more energy transfer out of the bath after 1 ps. On the other hand, increasing the energy transfer rate of the left bath does not cause any significant changes in the left bath energy and site 1 population. This may be due to the relatively low population at site 1, viz., after 1 ps, the populations at sites 1 and 4 are ≈0.11 and 0.28, respectively (see Figure 1).

### 4.4. Effect of Site Energy

**Figure 5:**
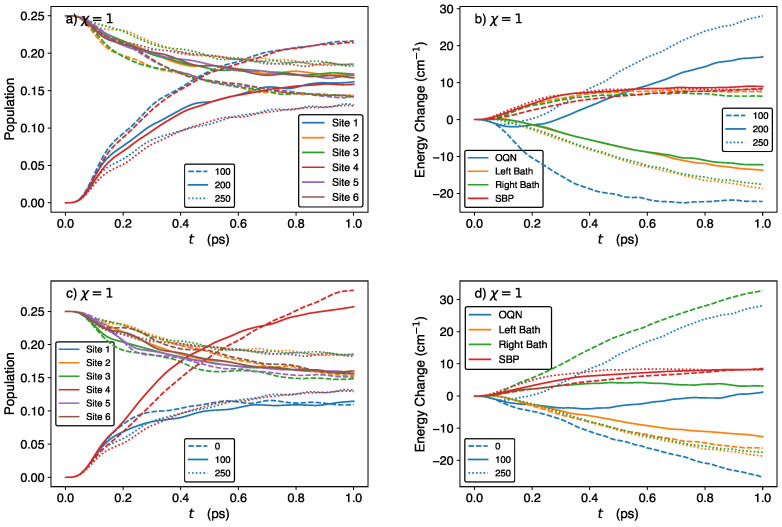
Time-dependent site populations (**left panels**) and energy changes (**right panels**) in the discharge phase (χ=1) for different SS energies. (**a**,**b**) Results for $E_{1}=E_{4}=100$ cm^−1^, $E_{1}=E_{4}=200$ cm^−1^, and $E_{1}=E_{4}=250$ cm^−1^. (**c**,**d**) Results for $E_{1}=250$ cm^−1^, and $E_{4}=0$, 100, and 250 cm^−1^. The values of the remaining parameters are $E_{i\in \{2,3,5,6\}}=200\;{\mathrm{cm}}^{-1}$, $T_{A}=T_{B}=300$ K, and $\lambda_{b}=35\;{\mathrm{cm}}^{-1}$.

We now investigate the effects of varying the SS energies on the site populations and energy changes. Time series and results after 1 ps for different combinations of the SS energies are shown in Figure 5 and Table S1 of the SI, respectively. As seen in Figure 5a, setting the SS energies equal to each other leads to roughly equal SS populations, with the SS populations decreasing when increasing from $E_{1}=E_{4}=100$ cm^−1^ to $E_{1}=E_{4}=250$ cm^−1^. When the energies of the SS are greater than those of the BSs, the BS are more populated than the SSs after 1 ps. Conversely, when the energies of the SS are lower than those of the BS, the SS are more populated than the BS after 1 ps. When the energies of the SS are equal to those of the BS, we see that the SS and BS populations approach each other over time, becoming almost equal after 1 ps. With regards to the energy changes (Figure 5b), setting the SS energies equal to each other leads to roughly equal changes in the left and right bath energies, with the bath energy changes decreasing and becoming more negative when increasing from $E_{1}=E_{4}=100$ cm^−1^ to $E_{1}=E_{4}=250$ cm^−1^. More specifically, there are energy gains in the baths for $E_{1}=E_{4}=100$ cm^−1^ and energy losses for the larger SS energies, with the loss increasing with increasing SS energy. Conversely, there is an energy loss from the system when $E_{1}=E_{4}=100$ cm^−1^ and energy gains for the larger SS energies, with the gain increasing with increasing SS energy. The SBP energy remains mostly unchanged for the different SS energies.

In Figure 5c,d, we plot the results for $E_{1}=250$ cm^−1^ and different values of $E_{4}$. As can be seen, increasing the energy of site 4 from 0 to 100 cm^−1^ does not cause a significant change in the site 4 population; however, increasing the energy of site 4 from 100 to 250 cm^−1^ causes a ≈50% drop in the site 4 population after 1 ps. Moreover, when the energy of site 4 is smaller than those of the remaining sites, site 4 becomes the most populated site after 1 ps. The populations of the remaining sites do not change significantly after going from $E_{4}=0$ to 100 cm^−1^, but they each increase by several percent after 1 ps after going from $E_{4}=100$ to 250 cm^−1^. As for the energy changes, increasing the energy of site 4 leads to more energy transfer from the right bath to the system, which in turn causes the OQN energy to change from decreasing to increasing. When both $E_{1}$ and $E_{4}$ are greater than $E_{BS}$ and the SS become populated, $\Delta E_{\mathrm{on}\text{-}\mathrm{site}}$ also becomes positive. Thus, when $\Delta E_{\mathrm{exch}}$ is also positive, the OQN will gain energy from the baths.

Based on the results above, a few general observations may be made. First, higher site energies are associated with lower site populations. In addition, for a higher site energy, more energy is transferred from the bath to the OQN. As seen in Figure 5, for the highest $E_{4}$, the (positive) OQN energy change increases and the (negative) bath energy changes decrease, while the magnitude of the SBP energy remains low and relatively constant, i.e., energy transfer from the bath to the OQN. If one changes the site energy of a particular site, then the population of that site will be mainly affected. If two sites have the same site energy, we expect them to eventually have equal populations. Finally, when all sites have the same site energy, all sites will have the same population after a sufficiently long period of time, despite starting with different initial populations (see Figure 5a).

## 5. Concluding Remarks

In this paper, we studied the population and energy transfer dynamics of an open quantum battery model, originally proposed in Ref. [18], over a wide range of parameter regimes. In the battery’s storage phase, we demonstrated that, in addition to no population leakage, there is no energy leakage from the battery into the attached baths. During the discharge phase, the changes in the populations and OQN energy are influenced by the bath temperatures, bath reorganization energies, and site energies. When increasing the temperature of one bath (while keeping the temperature of the other bath constant), we observed an increase in the energy transferred from that bath to the OQN. We found that the right bath (i.e., the bath connected to the exit site) exerts a larger influence on the exit site population than that exerted by the left bath (i.e., the bath connected to site 1) on the site 1 population. Moreover, when increasing the reorganization energy of the right bath, we observed an increase in the exit site’s population and a decrease in OQN energy. On the other hand, varying the reorganization energy of the left bath does not have pronounced effect on the population and energy. Regarding the site energies, when the energy of the exit site is lower than those of the BS, the OQN energy decreases in most of the parameter regimes studied. Lowering a given site energy causes the corresponding site population to increase. When the site energies are equal, the site populations reach roughly equal values after 1 ps, despite the different initial populations.

The results of our parameter space exploration show that different parameter sets render the QB conducive to different applications. In practice, this would amount to designing the QB in such a way that its properties are consistent with those of the desired parameter set. For example, for an energy battery, one may desire that the QB gains energy from its environment during the discharge phase. As we have shown, if one sets the site energies of the SS to be larger than those of the BS, then the QB gains energy as the SS populations grow in time. On the other hand, for an excitonic battery, one may desire to maximize the population of the exit site, regardless of the change in the OQN energy. In such a case, one could lower the exit site energy or lower the right-bath temperature while maintaining the left-bath temperature at its original value. If one would like to simultaneously reduce the loss in OQN energy and increase the exit site population, one could either increase the exit site energy or increase the left bath temperature. Overall, our findings shed light on design principles that could be used to construct different types of QBs operating between two thermal reservoirs.

## Data Availability

Data are contained within the article and supplementary materials.

## Supplementary Materials

The following supporting information can be downloaded at: https://www.mdpi.com/article/10.3390/molecules29040889/s1. Total energy drift, effect of varying surface site energies, time derivative of the bath and network-bath energies, and time-dependent quantum coherences.
