# Supplementary material for: Population and Energy Transfer Dynamics in an Open Excitonic Quantum Battery
Source: Molecules. 2024 Feb 17;29(4):889. doi: 10.3390/molecules29040889 (PMC10892759; doi:10.3390/molecules29040889)
Supplement: Supplementary file 1 [file molecules-29-00889-s001.zip › molecules-2823085-supplementary.pdf]

Supporting Information: Population and Energy Transfer Dynamics in an Open Excitonic Quantum Battery

Zhe Liu and Gabriel Hanna*

Department of Chemistry, University of Alberta, Edmonton, Alberta T6G 2G2, Canada

E-mail: gabriel.hanna@ualberta.ca

S1 Total energy drift

Figure S1 shows the average total energy versus time for the open QB during the discharge phase. As can be seen, the total energy drift is less than 1×10^{-2} cm^{-1}.

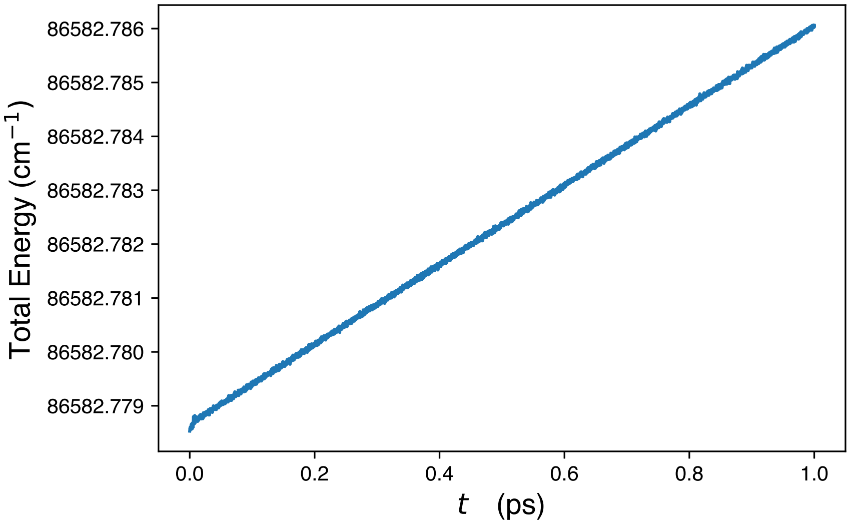

Figure S1: Average total energy as a function of time during the discharge phase. The average was calculated using an ensemble of 10,000 trajectories. All parameter values are the same as in the caption of Figure 1.

S2 Effect of varying surface site (SS) energies

Table S1: Site populations and energy changes during the discharge phase after 1 ps for different values of E_1 and E_4 with $E_{i\in\{2,3,5,6\}} = 200$ cm^{-1}.

E_n of SSs		Site Populations						Energy Changes [cm^{-1}]				
E_1	E_4	Site 1	Site 2	Site 3	Site 4	Site 5	Site 6	OQN	Left Bath	Right Bath	SBP	Exchange
250	0	0.1098	0.1528	0.1486	0.2818	0.1505	0.1565	-25.2871	-16.1494	32.8629	8.5810	25.5850
400	250	0.0549	0.1967	0.2050	0.1453	0.2033	0.1949	30.5251	-10.3941	-26.5583	6.4347	12.2778
100	100	0.2166	0.1441	0.1411	0.2139	0.1423	0.1419	-22.1795	7.5300	6.3110	8.3458	20.8624
200	200	0.1617	0.1698	0.1720	0.1584	0.1711	0.1670	16.9965	-13.7518	-12.1952	8.9579	16.9891
250	250	0.1312	0.1859	0.1848	0.1296	0.1857	0.1827	28.0908	-18.6962	-17.5048	8.1177	15.0413
200	50	0.1393	0.1472	0.1445	0.2703	0.1491	0.1496	-17.1695	-9.5391	18.8073	7.9086	23.3613
250	100	0.1146	0.1546	0.1570	0.2570	0.1565	0.1603	1.1991	-12.6415	3.1000	8.3498	21.1645
150	0	0.1725	0.1419	0.1381	0.2619	0.1410	0.1447	-34.9490	-4.2506	30.8475	8.3595	26.0402

S3 Time derivative of the bath and network-bath energies

To gain a better understanding of the energy changes during both the storage and discharge phases, we may derive an expression for the time derivative of the matrix element of the sum of the bath and network-bath energies, using the DECIDE equations of motion. For surface site n,

$$\begin{aligned}
\frac{d}{dt}[H_B + \hat{H}_{NB}]^{\beta\beta'} &= \frac{1}{2}\sum_j^M \frac{d}{dt}\left[P_{n,j}^2 + \omega_{n,j}^2 R_{n,j}^2 + \frac{C_{n,j}^2}{\omega_{n,j}^2}\hat{\mathcal{P}}_{nn} - C_{n,j}R_{n,j}\hat{\mathcal{P}}_{nn} - C_{n,j}\hat{\mathcal{P}}_{nn}R_{n,j}\right]^{\beta\beta'} \\
&= \frac{1}{2}\sum_j^M \left[P_{n,j}\frac{dP_{n,j}}{dt} + \frac{dP_{n,j}}{dt}P_{n,j} + \omega_{n,j}^2 R_{n,j}\frac{dR_{n,j}}{dt} + \omega_{n,j}^2\frac{dR_{n,j}}{dt}R_{n,j}\right. \\
&\quad + \frac{C_{n,j}^2}{\omega_{n,j}^2}\frac{d\hat{\mathcal{P}}_{nn}}{dt} - C_{n,j}\frac{dR_{n,j}}{dt}\hat{\mathcal{P}}_{nn} - C_{n,j}\hat{\mathcal{P}}_{nn}\frac{dR_{n,j}}{dt} \\
&\quad \left. - C_{n,j}R_{n,j}\frac{d\hat{\mathcal{P}}_{nn}}{dt} - C_{n,j}\frac{d\hat{\mathcal{P}}_{nn}}{dt}R_{n,j}\right]^{\beta\beta'} \\
&= \frac{1}{2}\sum_j^M \left[P_{n,j}\left(-\omega_{n,j}^2 R_{n,j} + C_{n,j}\hat{\mathcal{P}}_{nn}\right) + \left(-\omega_{n,j}^2 R_{n,j} + C_{n,j}\hat{\mathcal{P}}_{nn}\right)P_{n,j}\right. \\
&\quad + \omega_{n,j}^2 R_{n,j}P_{n,j} + \omega_{n,j}^2 P_{n,j}R_{n,j} + \frac{C_{n,j}^2}{\omega_{n,j}^2}\frac{d\hat{\mathcal{P}}_{nn}}{dt} \\
&\quad \left. - C_{n,j}P_{n,j}\hat{\mathcal{P}}_{nn} - C_{n,j}\hat{\mathcal{P}}_{nn}P_{n,j} - C_{n,j}R_{n,j}\frac{d\hat{\mathcal{P}}_{nn}}{dt} - C_{n,j}\frac{d\hat{\mathcal{P}}_{nn}}{dt}R_{n,j}\right]^{\beta\beta'} \\
&= \frac{1}{2}\sum_j \left[\frac{C_{n,j}^2}{\omega_{n,j}^2}\frac{d\hat{\mathcal{P}}_{nn}}{dt} - C_{n,j}R_{n,j}\frac{d\hat{\mathcal{P}}_{nn}}{dt} - C_{n,j}\frac{d\hat{\mathcal{P}}_{nn}}{dt}R_{n,j}\right]^{\beta\beta'} \qquad \text{(S1)}
\end{aligned}$$

To arrive at the third equality, we substituted the DECIDE equations of motion for the bath degrees of freedom (Eq. 9 of the main text). Based on this result, we can see that the expectation value $\frac{d}{dt}\langle H_B + \hat{H}_{NB}\rangle$ is 0 when $\frac{d}{dt}\langle\mathcal{P}_{11}\rangle$ and $\frac{d}{dt}\langle\mathcal{P}_{44}\rangle$ are 0. Thus, there is no energy transfer between the bath and OQN during the storage phase across all parameter regimes, even if a temperature gradient between the baths exists.

S4 Time-dependent quantum coherences

Figure S2 shows the time-dependent expectation values of the off-diagonal quantum coordinates (i.e., coherences) during the storage and discharge phases. During the storage phase,

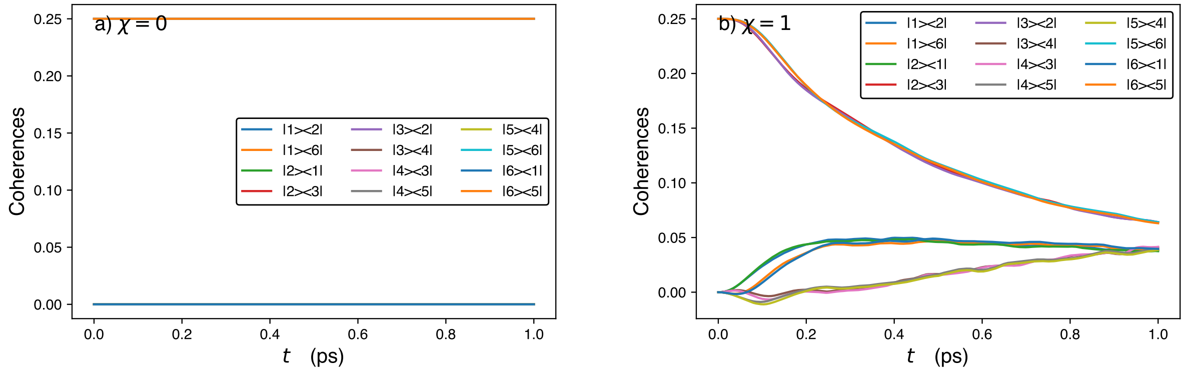

Figure S2: Time-dependence of the coherences during the storage (left panel) and discharge (right panel) phases. Ensemble averages were taken over 10,000 trajectories. The parameter set used to obtain these results is the same as that in the caption of Figure 1.

only $\langle\hat{\mathcal{P}}_{23}\rangle$, $\langle\hat{\mathcal{P}}_{32}\rangle$, $\langle\hat{\mathcal{P}}_{56}\rangle$, and $\langle\hat{\mathcal{P}}_{65}\rangle$ are non-zero (since the OQN is initialized in the dark state $|\psi_1\rangle$). During the discharge phase, their values decrease from 0.25 to ≈ 0.07 over the course of 1 ps. The remaining coherences start at zero and grow to ≈ 0.05 over the course of 1 ps.
